# Polyaniline/Tungsten Disulfide Composite for Room-Temperature NH_3_ Detection with Rapid Response and Low-PPM Sensitivity

**DOI:** 10.3390/s25133948

**Published:** 2025-06-25

**Authors:** Kuo Zhao, Yunbo Shi, Haodong Niu, Qinglong Chen, Jinzhou Liu, Bolun Tang, Canda Zheng

**Affiliations:** 1 School of Measurement and Control Technology and Communication Engineering, Harbin University of Science and Technology, Harbin 150080, China; iridescen7@163.com (K.Z.); jacksparrow23@163.com (H.N.); chenqinglong1008@yeah.net (Q.C.); liujinzhou0709@outlook.com (J.L.); tangbolun@sina.com (B.T.); zhengcanda@outlook.com (C.Z.); 2 China Higher Educational Key Laboratory for Measuring & Control Technology and Instrumentations of Heilongjiang Province, Harbin 150080, China

**Keywords:** polyaniline, conductive polymer gas sensing, WS_2_ nanosheet doping, heterojunction formation, flexible sensors

## Abstract

Polyaniline (PANI) is an important conductive-polymer gas-sensing material with working temperature and mechanical flexibilities superior to those of conventional metal oxide sensing materials. However, its applicability is limited by its low sensitivity, high detection limits, and long response/recovery times. In this study, we prepared PANI/WS_2_ composites via chemical oxidative polymerization and mechanical blending. A multilayer sensor structure—sequentially printed silver-paste heating electrodes, fluorene polyester insulating layer, silver interdigitated electrodes, and sensing material layer—was fabricated on a polyimide substrate via flexible microelectronic printing and systematically characterized using scanning electron microscopy, X-ray diffraction, and Fourier-transform infrared spectroscopy. The optimized 5 wt% WS_2_ composite showed enhanced gas-sensing performance, with 219.1% sensitivity to 100 ppm ammonia (2.4-fold higher than that of pure PANI) and reduced response and recovery times of 24 and 91 s, respectively (compared to 81 and 436 s for pure PANI, respectively). Notably, the PANI/WS_2_ sensor detected an ultralow ammonia concentration (100 ppb) with 0.104% sensitivity. The structural characterization and performance analysis results were used to deduce a mechanism for the enhanced sensing capability. These findings highlight the application potential of PANI/WS_2_ composites in flexible gas sensors and provide fundamental insights for PANI-based sensing materials research.

## 1. Introduction

Ammonia is a colorless, pungent, and lethal toxic gas. Its primary emission sources are industrial, agricultural, and vehicular exhaust. Through atmospheric circulation, ammonia generates ammonium salts that increase PM2.5 concentrations, posing a significant risk to health [1,2,3,4,5]. Acute exposure to high concentrations of ammonia causes severe physical harm and endangers human health. Prolonged exposure to ammonia gas concentrations exceeding 200 ppm can result in harmful respiratory irritation in adults and children, leading to headaches, nausea, and even life-threatening conditions. Furthermore, ammonia serves as a critical biomarker in medical diagnostics, particularly for liver diseases, *Helicobacter pylori*-related gastropathy, and kidney disorders. For instance, healthy individuals exhale ammonia at concentrations of approximately 0.3 ppm, while patients with renal dysfunction exhibit elevated levels averaging 0.8 ppm or higher [6,7]. Therefore, ammonia detection is vitally important to both environmental protection and medical diagnostics. Traditional gas analysis methods, such as gas chromatography and mass spectrometry, require bulky instruments, substantial labor, and time costs and suffer from result latency, making real-time monitoring challenging. Consequently, chemiresistive gas sensors with simple structures and real-time detection capabilities have attracted significant research attention. To date, numerous NH_3_ sensors have been developed, predominantly using metal oxides as the sensing materials. However, challenges persist: preparation processes are complex, costs are high, the sensors have limited flexibility, and power consumption is high owing to elevated operating temperatures. Thus, developing room-temperature ammonia sensors with high sensitivity, short response/recovery times, and flexible structures remains a crucial goal [8,9,10,11].

The selection of sensing materials is paramount in the development of high-performance gas sensors. Metal oxide semiconductors such as SnO_2_, WO_3_, and ZnO have been extensively applied to ammonia detection [12,13,14,15]. While these materials demonstrate good sensitivity, they typically require high operating temperatures (leading to high power consumption), exhibit poor selectivity, and lack the mechanical flexibility required for wearable applications. Recently, conductive polymers have gained increasing attention owing to their excellent physicochemical properties. Polyaniline (PANI) has emerged as a promising alternative for ammonia sensing [10,16,17]. PANI can be synthesized via simple chemical oxidative polymerization or electrochemical methods, and its conductivity can be tuned by adjusting oxidant types, reaction durations, and temperatures. Despite advantages like low operating temperatures and environmental stability, PANI-based sensors still suffer from inadequate sensitivity and slow response and recovery in ammonia detection. Modification is reportedly an effective method for enhancing the sensing performance of PANI. Representative modifiers include carbon-based materials and metal oxides. For example, Wan et al. [18] developed a flexible chemiresistive sensor using PANI/carbon nanotube (CNT) composites deposited on PET substrates, achieving responses to 30 to 100 ppm NH_3_ and a 1 ppm detection limit. Khuspe et al. [19] incorporated SnO_2_ nanoparticles into PANI via spin-coating, producing composite films with a 30% response to 100 ppm NH_3_ at room temperature (58% stability). These studies demonstrate that introducing carbon-based materials can provide additional conductive pathways, while the incorporation of n-type semiconductors, owing to the formation of p-n heterojunctions, significantly enhances the sensing performance of PANI.

With advancements in two-dimensional materials research, transition metal dichalcogenides (TMDs) have been shown to have significant potential in catalysis, lubrication, and optoelectronics applications. Compared to traditional metal oxides, TMDs exhibit unique 2D structures, larger specific surface areas, and higher charge carrier mobility, making them promising gas-sensing materials [20,21,22]. Zhao et al. [23] fabricated graphene/WS_2_ composite films with 2.42% and 1.73% responses to 100 ppm NH_3_ at 30 and 60 °C, respectively, and the same researchers validated the NH_3_ sensing capability of WS_2_ using first-principles calculations. Zhang et al. [24] synthesized MoS_2_/WS_2_ heterojunctions via hydrothermal methods, achieving a remarkable 236% response to 500 ppm NH_3_, a 20-ppm detection limit, and 2.6 s recovery time. Sharma et al. [25] developed WS_2_ nanosheet-based ammonia sensors with response and recovery times of 54 and 66 s, respectively, to 5 ppm NH_3_. These results highlight the advantages of WS_2_ in terms of its carrier mobility and adsorption capacity. However, the low intrinsic conductivity of this material and its poor mechanical flexibility limit its applicability in the design of flexible devices. Although conductive polymers like PANI exhibit excellent compatibility with flexible substrates, their sensitivity and response/recovery performance are unsatisfactory. These facts, as well as the fact that there are limited reports on PANI-TMD composites, necessitate further exploration in this field.

To address these challenges, in this study, we leveraged the complementary properties of PANI and WS_2_ to fabricate a flexible multilayer ammonia sensor using PANI/WS_2_ as the sensing material. PANI/WS_2_ composites were prepared using chemical oxidative polymerization and solution blending. A polyimide substrate was employed to construct the sensor architecture via layer-by-layer printing using a flexible microelectronic printer, incorporating fluorene polyester (FPE) insulation, conductive silver paste heating layers, and interdigitated electrodes. The morphology and structure of the composite were characterized using scanning electron microscopy (SEM), X-ray diffraction (XRD), and Fourier-transform infrared spectroscopy (FTIR). The sensing material was subsequently coated onto the interdigitated electrodes via dispensing printing to complete the flexible sensor. A gas-sensing test platform was used to evaluate the performance of the sensor across NH_3_ concentrations in the range of 0.1–100 ppm. Finally, the sensing mechanism was elucidated based on the characterization and sensing performance results, highlighting a simple, eco-friendly methodology for future wearable gas detection applications.

## 2. Experimental Section

### 2.1. Materials

All chemicals were used as received unless otherwise specified. Aniline (C_6_H_5_NH_2_, analytical reagent (AR) grade; Macklin Biochemical Co., Ltd., Shanghai, China) was distilled at 200 °C under reduced pressure prior to use and stored at low temperature. Ammonium persulfate ((NH_4_)_2_S_2_O_8_, AR grade), turpentine oil (C_10_H_18_O, AR grade), tungsten disulfide (WS_2_), hydrochloric acid (HCl), *N*-methylpyrrolidone (NMP, C_5_H_9_NO, CAS: 67-44-5, purity ≥99.9%), *N*,*N*-dimethylformamide (DMF, CAS: 68-12-2, purity ≥99.9%), and ethanol (C_2_H_5_OH, CAS: 64-17-5, purity ≥99.8%) were obtained from Fuchen Chemical Reagent Co., Ltd. (Tianjin, China) and Aladdin Reagent Co., Ltd. (Shanghai, China). Silver conductive paste was procured from Shenzhen Saiya Electronic Paste Co., Ltd. (Shenzhen, China).

### 2.2. Fabrication of PANI/WS_2_ Sensing Films

**Figure 1:**
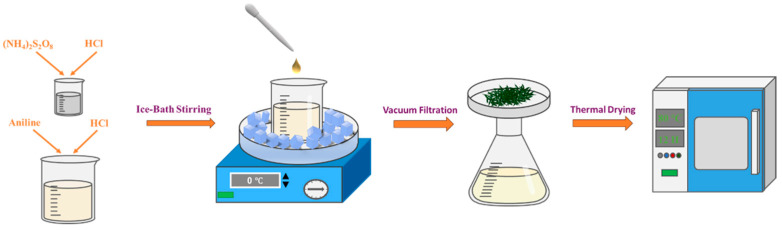
Schematic of polyaniline (PANI) preparation via chemical oxidative polymerization.

Figure 1 illustrates the synthesis process of PANI. The synthesis was initiated by dissolving distilled aniline (5 mL) in hydrochloric acid (HCl; 1 M, 500 mL) under magnetic stirring for 2 h to form a homogeneous aniline hydrochloride solution. In parallel, ammonium persulfate (11.4 g) was dissolved in HCl (1 M, 50 mL) with continuous agitation for 2 h to obtain the oxidant solution. The aniline solution was subsequently cooled in an ice bath, and the ammonium persulfate oxidant solution was introduced to it dropwise via a burette over a period of 1 h under vigorous stirring. The polymerization was allowed to proceed in the ice bath for 8 h, after which the dark green suspension was vacuum-filtered and the precipitate subjected to five consecutive washing cycles, with 0.1 M HCl and absolute ethanol alternately used as the solvent for washing, to remove residual impurities. Finally, after drying in a vacuum oven at 80 °C for 12 h, the purified PANI was yielded as dark green conductive particles.

**Figure 2:**
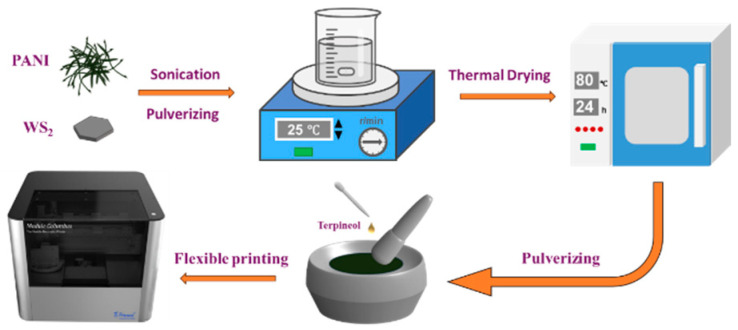
Fabrication process of PANI/WS_2_ composite sensing material.

Following the drying process, the PANI was combined with tungsten disulfide (WS_2_) nanosheets via a solution blending method (Figure 2). Precisely weighed PANI and WS_2_ (at a pre-determined mass ratio) were individually ground in an agate mortar for 1 h. The PANI powder was then dispersed in *N*,*N*-dimethylformamide (DMF; 5 mL) and subjected to sequential ultrasonication (40 kHz, 100 W) and magnetic stirring (500 rpm), each for 1 h. The ground WS_2_ nanosheets were then added to the PANI/DMF suspension, and this was followed by an additional cycle of ultrasonication and magnetic stirring (1 h each) to ensure homogeneous mixing. The mixture was dried in an oven at 80 °C for 24 h, after which the composite was re-ground for 1 h in a mortar with terpineol (2 mL) added dropwise. This final grinding step produced a uniform composite slurry with suitable rheological properties for direct ink writing using a microelectronic dispenser printer.

### 2.3. Characterization

The microstructural morphology of PANI and the PANI/WS_2_ composites was examined using field-emission scanning electron microscopy (FESEM; ZEISS Gemini SEM 300, Oberkochen, Germany) at accelerating voltages in the range of 3–15 kV. Crystal structure analysis was performed based on X-ray diffraction (XRD, Rigaku SmartLab 3 kW, Tokyo, Japan) with Cu-Kα radiation (*λ* = 1.5406 Å); the 2*θ* range was 20–80° and the scan rate 2° min^−1^. Functional group evolution was investigated using Fourier-transform infrared spectroscopy (FTIR, PerkinElmer Frontier 100, Waltham, MA, USA) in attenuated total reflectance (ATR) mode, with spectral acquisition spanning 400–4000 cm^−1^ at a resolution of 4 cm^−1^ (32 scans per measurement).

### 2.4. Design and Fabrication of Sensor Structure and Construction of Gas-Sensing Test Platform

**Figure 3:**
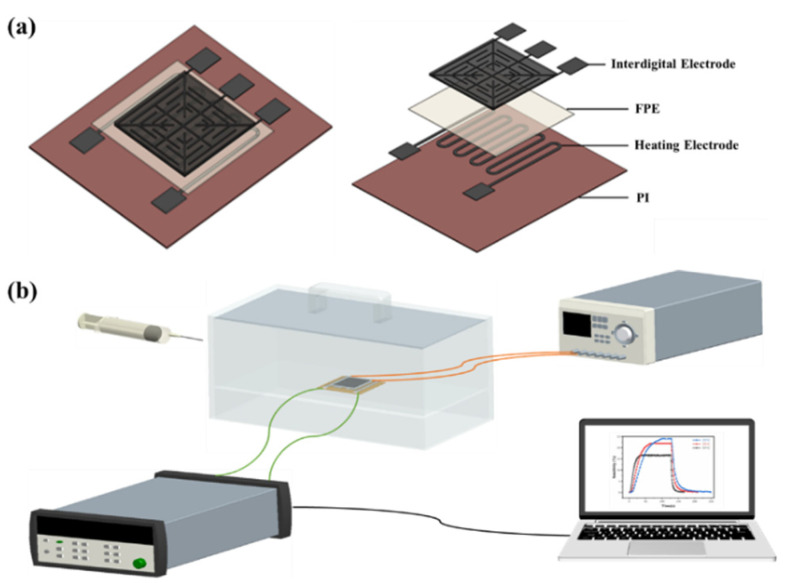
Sensor structure and gas-sensing test platform.

We manufactured flexible gas sensors using a microelectronic distributor printer (Scientific 3, Zhongbin Technology Co., Ltd., Shanghai, China). The device structure consisted of four sequentially stacked layers on a polyimide substrate (Figure 3a): a flexible printed silver heating electrode, an FPE insulation layer, a silver forked electrode (IDE), and a composite sensing-material coating. To form the insulation layer, a homogeneous solution was prepared by dissolving FPE particles (0.4 g) in 4 mL of NMP, followed by sonicating the mixture for 2 h and magnetically stirring it for 3 h. Next, the coating module of the printer was used to coat the FPE solution blade onto the heating electrode (avoiding contact pads), and it was subsequently cured in a vacuum oven at 80 °C for 24 h. After patterning the IDE, the PANI/WS_2_ composite slurry (prepared using the method described in Section 2.2) was evenly distributed onto the IDE and dried at 80 °C for 12 h. The completed sensor was aged at 30 °C for 5 days to stabilize its performance.

The gas-sensing test platform comprised a sealed chamber connected to a DC power supply, data acquisition system, and computer (Figure 3b). Specific concentrations of ammonia gas were injected into the chamber using a gas-tight syringe. The heating electrode maintained the sensor at ambient temperature during testing. Resistance changes induced by the interaction of ammonia with the sensing layer were recorded via the IDE and analyzed using dedicated software to extract the response/recovery parameters. The physical diagram of the sensor structure is shown in Figure S1.

## 3. Results and Discussion

### 3.1. Characterization of Composite Materials

**Figure 4:**
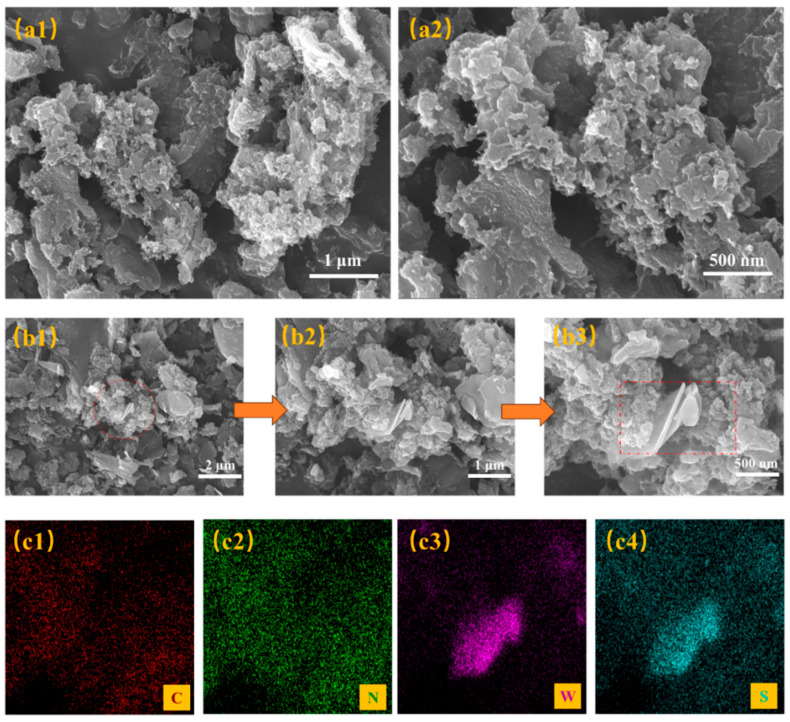
Scanning electron microscopy (SEM) images of (**a1**,**a2**) PANI and (**b1**–**b3**) of 5 wt% WS_2_ PANI/WS_2_ composite. (**c1**–**c4**) Energy-dispersive spectroscopy (EDS) elemental maps of the 5 wt% WS_2_ PANI/WS_2_ composite.

Figure 4(a1,a2) display scanning electron microscopy (SEM) images of pristine PANI at 1 μm and 500 nm magnifications. The as-synthesized PANI sample is characterized by irregular particle agglomeration and minimum particle dimensions of approximately 80 nm. Figure 4(b1–b3) show SEM images of the PANI/WS_2_ (5 wt%) composite at different scales. The solution blending method, coupled with ultrasonication and magnetic stirring, achieved enhanced dispersion homogeneity. Pure PANI exhibits an irregular aggregation state (Figure 4(a1,a2)), with a minimum particle size of approximately 80 nm, and pores with sizes of the order of micrometers on the surface. In the 5 wt% WS_2_ composite (Figure 4(b1–b3)), WS_2_ nanosheets are embedded into the PANI matrix, forming a micro/nano multilevel pore structure with an increased specific surface area. Notably, increasing the WS_2_ content to 7 wt% may lead to agglomeration (see Supplementary Materials, Figure S3), which may cause pore blockage and deteriorate the sensing performance. Figure 4(c1–c4) shows energy-dispersive spectroscopy (EDS) elemental maps of the 5 wt% WS_2_ composite, confirming the uniformity of the PANI distribution and successful WS_2_ incorporation within the composite, both of which are expected to lead to improved gas sensing capabilities. The cross-sectional image of the sensitive film is shown in Figure S2.

**Figure 5:**
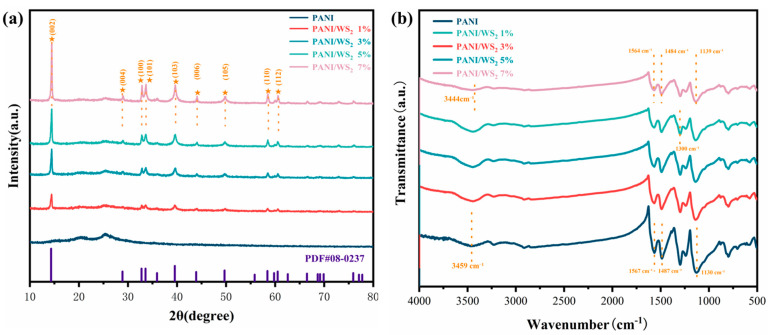
(**a**) X-ray diffraction (XRD) and (**b**) Fourier-transform infrared (FTIR) spectra of the composite material.

The XRD patterns of the different PANI/WS_2_ composites (Figure 5a) reveal a distinct structural evolution trend. The XRD pattern of pristine PANI features a broad amorphous halo at 20–30° (2*θ*) that is characteristic of the disordered polymer chains. With the incorporation of WS_2_ into the material, sharp diffraction peaks emerge at 14.4°, 29°, 32°, 33°, 39°, 44°, 49°, 58°, and 60° that are assignable to the (002), (004), (100), (101), (103), (006), (105), (110), and (112) crystallographic planes, respectively, of hexagonal WS_2_ (JCPDS 08-0237). In the XRD profiles, the position of the characteristic peak of WS_2_ (such as 14.4° (002)) in the composite material is consistent with that of pure WS_2_, indicating the absence of lattice distortion. The intensity of the amorphous dispersion peak of PANI decreases with increasing WS_2_ content, indicating that WS_2_ restricts the movement of PANI segments [26,27,28].

FTIR analysis (Figure 5b) allowed us to evaluate the molecular-level interactions in the composites. The broad N–H stretching vibration, at ~3400 cm^−1^ in pure PANI, gradually broadens with WS_2_ loading, suggesting that the hydrogen bonding was strengthened. The characteristic C=C stretching vibrations of benzenoid (1487 cm^−1^) and quinoid (1567 cm^−1^) rings undergo 5–8 cm^−1^ red shifts, which are indicative of enhanced π-π stacking between WS_2_ and the conjugated PANI backbone. The C=C bond redshift (benzene ring 1487 → 1482 cm^−1^, quinone ring 1567 → 1562 cm^−1^) indicates that the PANI aromatic ring forms π–π stacking with the WS_2_ sulfur plane in a face-to-face configuration, with a spacing of ~0.34 nm (typical π stacking distance) accommodated by the (002) interplanar spacing (0.62 nm) of layered WS_2_. The invariance of the in-plane C–H bending vibration at 1130 cm^−1^ confirms that the planarity of the polymer backbone is preserved. The FTIR spectra of samples, containing ≤3 wt% WS_2_, show only minor changes in peak intensities (<5%), and peak-shift or new peaks are not observed. This result indicates the absence of strong chemical bonds and suggests that van der Waals forces dominate the interactions. [29,30,31].

### 3.2. Sensor Performance Testing

The gas sensing performance of the prepared composite sensors with WS_2_ mass fractions of 0, 1, 3, 5, and 7 wt% was evaluated. Each sensor was placed in a gas chamber and tested under static conditions by sequentially exposing it to gaseous ammonia at concentrations of 1, 5, 10, 20, 50, and 100 ppm. The temperature was maintained at 25 ± 2 °C, and the relative humidity was 37 ± 3%. The sensitivity of the sensor was defined in terms of the sensor response as follows:

(1) $$Response\;\left(\%\right)=\frac{R_{g}-R_{a}}{R_{a}}\times 100\%$$

where *R_g_* is the resistance of the sensor in ammonia gas and *R_a_* is the baseline resistance, i.e., the resistance in clean air.

**Figure 6:**
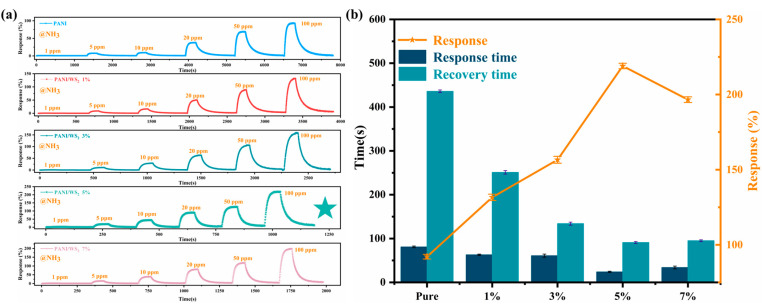
(**a**) Response to different concentrations of ammonia of sensors made from PANI/WS_2_ composites with different WS_2_ contents. (**b**) Sensitivity, response time, and recovery time versus WS_2_ content of the composite in the sensor.

The sensitivity curves of sensors with different compositions, shown in Figure 6a, indicate that the response to ammonia is concentration dependent. All the composites exhibit p-type semiconductor behavior when exposed to the reducing gas. The pure PANI sensor (0 wt% WS_2_) shows the lowest sensitivity (92.1% at 100 ppm), a prolonged response time (81 s), and a very prolonged recovery time (436 s), which significantly limit its practical applicability.

With increasing WS_2_ content over the range of 0–5 wt%, both the sensitivity and the response/recovery times progressively improve. The best performance was realized using the 5 wt% composite: 219.1% sensitivity at 100 ppm NH_3_, representing a 2.38-fold enhancement over that of pure PANI, along with a 3.3-fold shorter response time (24 s) and 4.8-fold shorter recovery time (91 s). However, at 7 wt% WS_2_, the sensitivity dropped to 196%, and the recovery was slower (95 s), which were attributed to a reduction in surface porosity owing to nanosheet agglomeration (Figure 6b).

**Figure 7:**
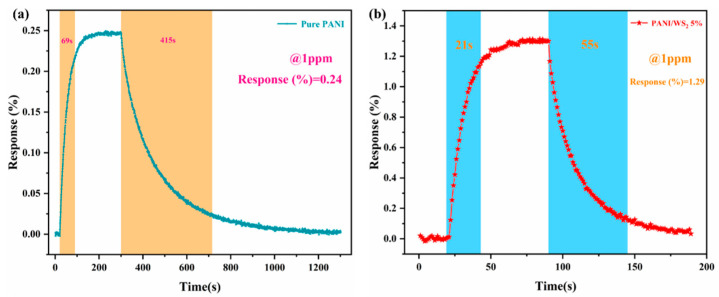
Response-recovery curves of (**a**) pure PANI and (**b**) 5% WS_2_ composite sensor at 1 ppm NH_3_.

To investigate the gas sensing capabilities at trace levels, the responses of the pure PANI and PANI/WS_2_ (5 wt%) sensors were compared at 1 ppm NH_3_ (Figure 7a,b). The pure PANI sensor exhibited a response value of 0.24% with long response and recovery times (69 and 415 s, respectively). In contrast, the PANI/WS_2_ (5 wt%) composite demonstrated significantly enhanced performance: a 5.37-fold higher response (1.29%), 3.28-fold shorter response time (21 s), and 7.54-fold shorter recovery time (55 s). This improvement is attributed to the hierarchical porosity of the composite material and WS_2_-induced heterojunction formation, which synergistically enhance gas adsorption kinetics and charge transfer efficiency.

**Figure 8:**
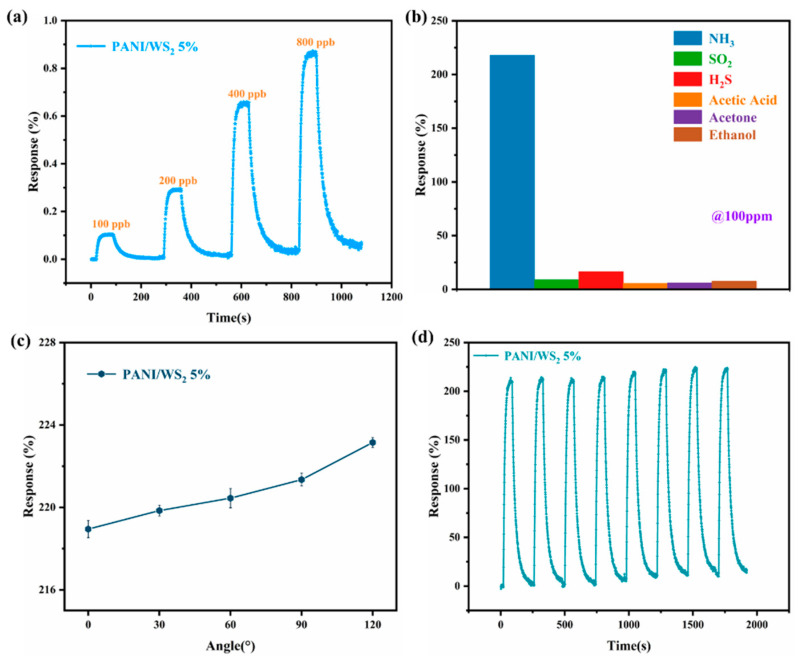
Ammonia sensing performance characterization of PANI/WS_2_ (5 wt% WS_2_) composite sensor. (**a**) Low-concentration response curves. (**b**) Selectivity, (**c**) bending angle, and (**d**) repeatability test results at 100 ppm NH_3_.

The trace-level detection capability of the PANI/WS_2_ (5 wt% WS_2_) composite sensor was evaluated (Figure 8a). At 100 ppb ammonia, the sensor exhibited a detectable response of 0.104%, which increased to 0.871% at 800 ppb. Although a slight baseline drift was observed below 1 ppm, stable sensing, within an acceptable range, was maintained. Selectivity testing of the same sensor (Figure 8b) revealed distinct responses to 100 ppm of various gases, NH_3_ (218%), SO_2_ (9%), H_2_S (16.5%), acetic acid (5.54%), acetone (6%), and ethanol (7.5%), confirming the excellent ammonia selectivity of the sensor. Mechanical flexibility tests (Figure 8c) demonstrated consistent performance under bending stress. At bending angles of 30°, 60°, 90°, 120°, and 150°, the sensor showed average responses of 218.95%, 219.85%, 220.45%, 221.35%, and 223.15%, respectively, to 100 ppm NH_3_. The marginal response increase with larger angles may arise from tensile strain-induced surface area expansion in the composite. Repeatability testing (Figure 8d) over eight cycles at 100 ppm NH_3_ yielded an average response of 219% (RSD = 1.2%), confirming that the operational stability of the composite sensor was excellent.

**Figure 9:**
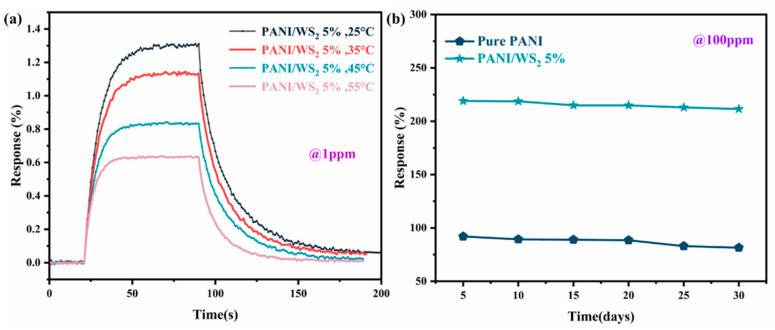
(**a**) Gas sensing performance at various temperatures. (**b**) Long-term stability results.

The PANI/WS_2_ (5 wt%) composite sensor was tested at different temperatures (25, 35, 45, and 55 °C) in 1 ppm NH_3_ atmosphere (Figure 9a). The sensitivity decreased from 1.29% to 0.63%, while response times were shortened from 21 s to 12 s and recovery times from 55 s to 31 s as the temperature was increased over this range. This trend is attributed to the enhanced thermal motion of NH_3_ molecules at higher temperatures, which reduces adsorption stability but accelerates desorption kinetics. The sensor maintained reliable detection at 1 ppm NH_3_ over this temperature range from room temperature to 55 °C, suggesting that it could have the desired flexibility characteristics for applications requiring rapid responses when integrated with heating modules. Humidity variation in the range of 35–75% RH produced minimal changes in the response (218.83–220.11%) to 100 ppm NH_3_ (Figure S4). A 30-day long-term stability assessment result showed that the sensitivity of the composite sensor decreased by 3%, while that of the pure PANI sensor decreased by 11.4%. (Figure 9b). Comparing the results obtained in this study with those reported in the literature, the advantages of the composite in terms of its sensitivity, lower detection limit, and shorter response/recovery times are apparent (Table 1).

### 3.3. NH_3_ Sensing Mechanism of PANI/WS_2_

Although NH_3_ exhibits reducibility, its deprotonation dominates the response in the PANI system: NH_3_ binds to H^+^ to reduce the concentration of polarons, leading to an increase in resistance (p-type behavior). The n-type characteristic of WS_2_ amplifies this effect through the heterojunction. The intrinsic insulator PANI undergoes protonation in the presence of dilute hydrochloric acid during synthesis. The acidic environment facilitates protonation at the quinoid ring nitrogen atoms, converting quinoid segments into benzenoid structures. This structural rearrangement produces p-type semiconductor behavior under an external electric field owing to hole-dominated charge transport. When exposed to the alkali ammonia (NH_3_), the conductive PANI matrix is deprotonated, reducing the hole concentration and increasing the electrical resistance of the material. Conversely, upon NH_3_ removal, ammonium ions (NH_4_^+^) decompose into NH_3_ and protons (H^+^), and the conductivity of PANI is restored. The reversible reaction mechanism is expressed as follows [36,37,38]:

(2) PANI⁺+NH₃→PANI+NH₄⁺

**Figure 10:**
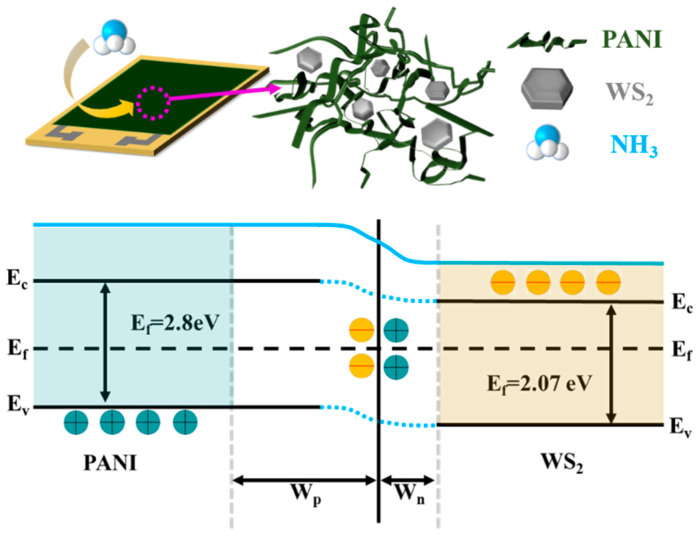
Ammonia sensing mechanism of the sensor.

As demonstrated by the results shown in Figure 8 and discussed above, the introduction of WS_2_ nanosheets into PANI significantly improves sensor performance. First, a p-n heterojunction is formed at the interface between p-type PANI and n-type WS_2_. According to previous studies, PANI has a bandgap of 2.8 eV (p-type semiconductor), while that of WS_2_ is 2.07 eV (n-type semiconductor). As shown in Figure 10, in the p-n heterojunction, electrons and holes move in opposite directions at the interface, forming a depletion layer. As carriers migrate, the Fermi level eventually equilibrates. The incorporation of WS_2_ introduces a higher potential barrier, resulting in increased resistance for the composite compared to that of pure PANI.

In the presence of ammonia, NH_3_ molecules neutralize protons in conductive PANI to form NH_4_^+^. Simultaneously, the depletion layer at the heterojunction interface provides additional holes for interaction with NH_3_ molecules, enhancing sensitivity. Furthermore, the SEM images of the composites reveal that the embedded WS_2_ nanosheets increase the PANI dispersion, leading to a more uniform distribution. The enhanced dispersion results in greater porosity, and the two-dimensional sheet structure provides a larger specific surface area. Compared to pure PANI, the composite offers more adsorption sites and therefore faster response and recovery. In summary, appropriate WS_2_ doping optimizes the gas sensing performance of PANI/WS_2_ composites.

## 4. Conclusions

In this study, a flexible ammonia sensor operable at room temperature based on a PANI/WS_2_ composite was realized. The composites were synthesized via chemical oxidative polymerization and mechanical blending, making use of microelectronic printing technology. The optimized composite with 5 wt% WS_2_ demonstrated significantly enhanced gas sensing performance: the sensitivity was 219.1% at 100 ppm NH_3_ (a 2.38-fold improvement over that of pure PANI), with response and recovery times reduced to 24 and 91 s (3.3- and 4.8-fold shorter, respectively, than the times for pure PANI). The sensor exhibited detectable responses to 100 ppb NH_3_ (0.104%), along with exceptional selectivity (an NH_3_ response 24–39 times higher than interferent gas responses), mechanical flexibility (stable sensitivity during 120° bending), and long-term stability (a 3% sensitivity drop over 30 days). Mechanistic studies revealed that p-n heterojunction formation owing to WS_2_ incorporation enhances carrier migration and surface adsorption, while the two-dimensional structure of WS_2_ improves PANI dispersion and porosity, synergistically boosting the sensitivity and response kinetics of the sensor. This work provides a novel general strategy for the development of highly sensitive, low-power flexible gas sensors for wearable healthcare monitoring and environmental detection applications based on heterojunction interface optimization or hybridization with 2D materials.

## Figures and Tables

**Table 1:** Performance of several common types of NH_3_ gas sensor.

Material	Detection Limit	Temperature	Concentration	Response	Response/Recovery Time	Reference
PANI		RT	100 ppm	96%	122 s/1235 s	[32]
PANI/WO_3_	3 ppm	RT	100 ppm	150%	122 s/165 s	[33]
PANI/Ti_3_C_2_T*_X_*	5 ppm	RT	20 ppm	55.9%	40 s/200 s	[34]
PANI/MWCNTs	33 ppm	RT	50 ppm	117%	47 s/~ s	[35]
rGO-PANI	1 ppm	RT	100 ppm	6.2%	219 s/541 s	[36]
PANI/WS_2_	0.1 ppm	RT	100 ppm	219.1%	24 s/91 s	This work

## Data Availability

The original contributions presented in this study are included in the article. Further inquiries can be directed to the corresponding author.

## Supplementary Materials

The following supporting information can be downloaded at https://www.mdpi.com/article/10.3390/s25133948/s1: Figure S1: Physical structure diagram of flexible gas sensor; Figure S2: Cross section of sensitive film; Figure S3: Scanning electron microscopy (SEM) image of PANI/WS_2_ composite with 7 wt% WS_2_ loading; Figure S4: Humidity-dependent sensitivity of 5 wt% PANI/WS_2_ composite sensor exposed to 100 ppm NH_3_.
