# Supplementary material for: Polyaniline/Tungsten Disulfide Composite for Room-Temperature NH3 Detection with Rapid Response and Low-PPM Sensitivity
Source: Sensors (Basel). 2025 Jun 25;25(13):3948. doi: 10.3390/s25133948 (PMC12251670; doi:10.3390/s25133948)
Supplement: Supplementary file 1 [file sensors-25-03948-s001.zip › sensors-3685224-supplementary.pdf]

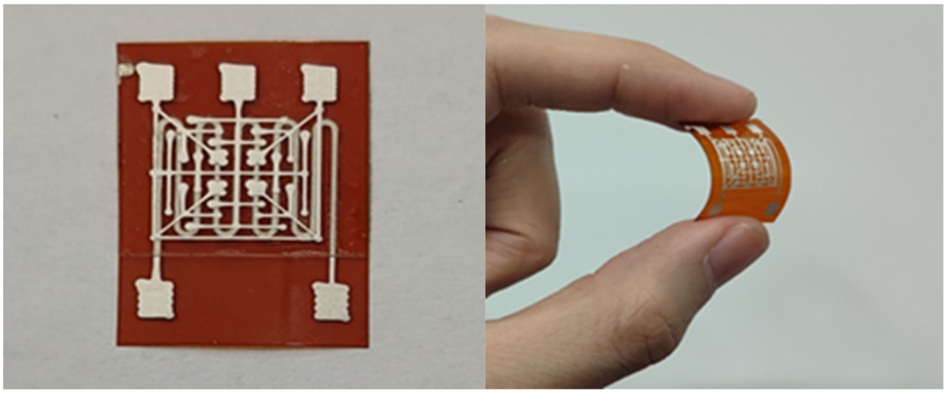

Figure S1. Physical structure diagram of flexible gas sensor

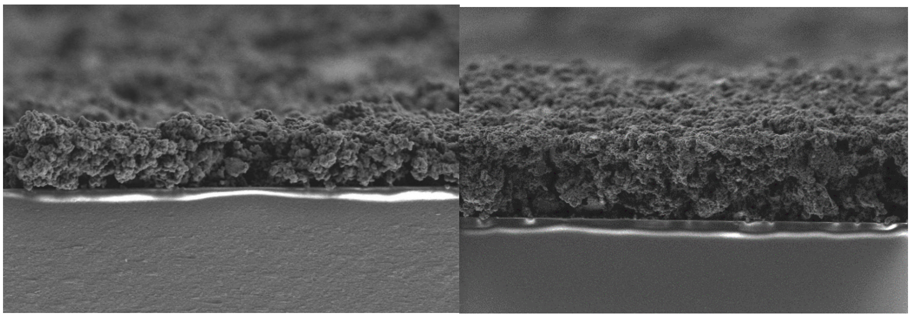

Figure S2 .Cross section of sensitive film

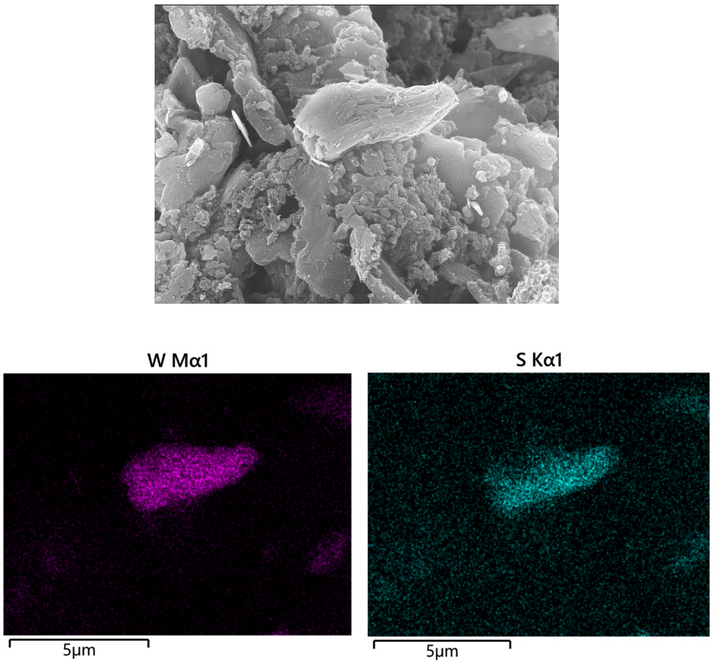

Figure S3.Scanning electron microscopy (SEM) image of PANI/WS_2 composite with 7 wt% WS_2 loading

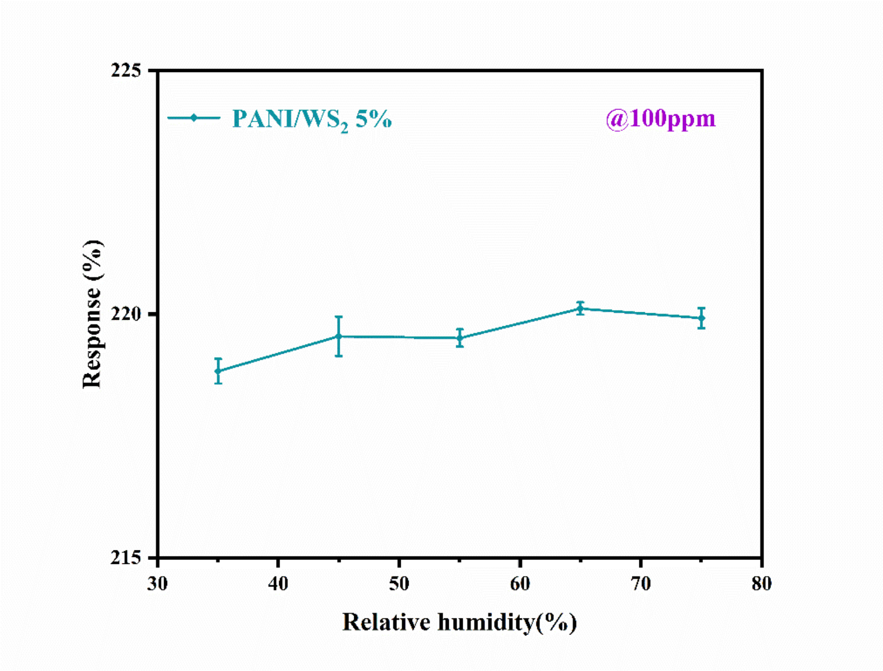

Figure S4. Humidity-dependent sensitivity of 5 wt% PANI/WS_2 composite sensor exposed to 100 ppm NH_3
